# Mixed valence mono- and hetero-metallic grid catenanes†
†Electronic supplementary information (ESI) available: Details of the CSD database search, additional crystallographic information on **3–6** with relevant tables, EDS analysis and EDS plots. CCDC 1054509–1054512. For ESI and crystallographic data in CIF or other electronic format see DOI: 10.1039/c5sc01851j



**DOI:** 10.1039/c5sc01851j

**Published:** 2015-06-30

**Authors:** Chandan Giri, Filip Topić, Massimo Cametti, Kari Rissanen

**Affiliations:** a University of Jyvaskyla , Department of Chemistry , Nanoscience Center , P. O. Box. 35, FI-40014 University of Jyvaskyla , Finland . Email: kari.t.rissanen@jyu.fi; b Department of Chemistry , Materials and Chemical Engineering “Giulio Natta” , Politecnico di Milano , Via L. Mancinelli 7 , 20131 Milano , Italy . Email: massimo.cametti@polimi.it

## Abstract

Here, we report on the multicomponent self-assembly and single crystal X-ray diffraction study of a series of three interlocked mixed valence mono- and hetero-metallic [2]-catenanes made of [2 × 2] metallo-grids. They show unique structural features and highlight the essential roles of both the Cu(ii)/Cu(i) pair and of the conformationally adaptable organic ligands for achieving catenation of grids.

## Abstract 2

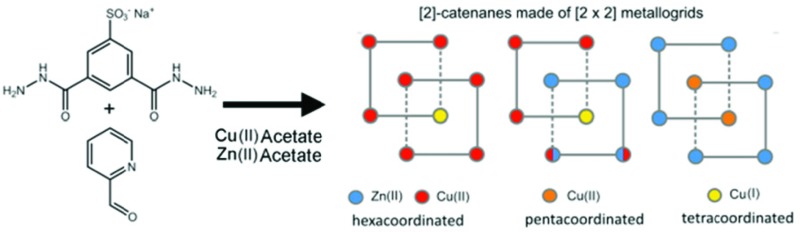
Multicomponent self-assembly was employed to obtain, in the solid state, a series of mixed valence mono- and hetero-metallic grid catenanes, which were characterized by single crystal X-ray diffraction.

## Introduction

In the last half century, the scientific community has heavily focused on the design and synthesis of structurally demanding molecular architectures following the desire for complexity, aesthetic beauty, topological novelty and, most importantly, novel emerging properties.^1^ To this day, differently interlocked and/or entangled species, such as rotaxanes,^1*a*^ catenanes,^1j,k^ knots^1*h*^ and other complex structures^1c,d,f,i^ have been vigorously pursued. In particular, catenanes^3^ consist of two or more mechanically interlocked macro- or metallocycles, which cannot be separated without breaking a bond. The first discovery of a metal complex [2]-catenane self-assembled in one step was reported by Fujita and co-workers in 1994,^4^ yet exploration into this new field remained mostly limited to [M_2_L_2_]_2_ [2]-catenane species (M = metal, L = ligand). Only very recently have some examples of more complex catenanes been reported.^5^ For instance, Clever *et al.* reported on a dimeric [M_2_L_4_]_2_ interpenetrated coordination cage, which forms by the assembly of Pd(ii) metal ions with a simple ditopic pyridyl ligand.^5*a*^ Notably, the same group, by using a very similar but slightly shorter ligand, was able to achieve double ([M_2_L_4_]_2_) and triple ([M_2_L_2_]_3_) catenation in a stepwise process triggered by halide anions.^5*b*^ A fully organic [3]-catenane was, instead, obtained by Nitschke *et al.*, by employing an elegant but intricate self-assembly procedure based on a metal ion template and utilizing five different precursor molecules.^5*c*^ An increase in the number of catenated species can be achieved, as demonstrated in the dynamic systems comprised of poly-catenanes (up to [7]-catenane) based on a tetrahedrally-shaped cage framework.^5*d*^ Leigh *et al.* have recently demonstrated the preparation of a Solomon link,^5*e*^ a pentafoil knot^5*f*^ and a Star of David catenane^5*g*^ in one^5e,f^ or two steps,^5*g*^ through careful ligand design while also taking advantage of iron(ii) and anion (chloride^5*f*^ or sulfate^5*g*^) templating effects. Finally, the assembly of a two-dimensional metal–organic network possessing Borromean links has recently been reported by Hardie *et al.* ^5*h*^

In the pursuit of the construction of chemical objects of considerable structural complexity, multicomponent self-assembly has been proven to be a very successful technique,^6^ as demonstrated by many of the examples described above. It relies on the designed organization of carefully selected sub-components, which are linked together through the simultaneous formation of covalent and/or coordinative bonds, ultimately yielding the desired superstructure.

An assembly constituted by arrays of linear molecules held together and shaped into a discrete crisscrossed two-dimensionally ordered framework is usually termed a molecular grid or, when metal ions interconnect a set of organic ligands, a metallogrid.^1*e*^ Although grid-like systems^7^ have become relatively common over the last decade,^1*e*^ and their potential for applications has been demonstrated,^1i,t,2^ a higher-level organization of grids as components remains quite rare.^1e,g^ In the context of interlocked systems, only two notable examples involving metallogrids can be found, namely that of the so far only homometallic grid catenane by Thompson *et al.* ^14^ and the recent elegant use of a metallogrid as a scaffold in the synthesis of a Solomon link, reported by Leigh *et al.* ^1*b*^ To the best of our knowledge, no examples of catenanes formed by heterometallic grids^7a,1m,n^ have been reported to this date.

**Scheme 1:**
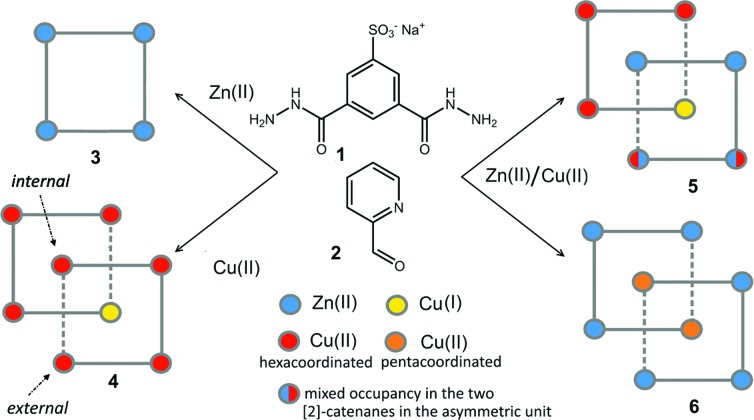
The chemical formulae of components **1** and **2** and a schematic representation of grid **3** and the [2]-catenanes **4–6**.

Here, we report on the isolation in the solid state of three [2]-catenane species where the macrocyclic ring units are made of [2 × 2] metal grids. These metal–organic architectures are formally created by the self-assembly of 32 components (8 × **1**, 16 × **2** and 8 × metal, with **2** = 2-formylpyridine and metal = Zn(ii) or Cu(ii)/(i)) and they spontaneously precipitate as crystalline solids from a H_2_O/DMF mixture. The first species, **4** (Scheme 1), is a mono-metallic mixed valence Cu(ii)/Cu(i) grid catenane, while the other two, **5** and **6** (Scheme 1), are bi-metallic Cu/Zn complexes. Among the eight metal ions that constitute the nodes of the two concatenated grid units, six of them can be considered as external while the remaining two can be viewed as internal with respect to the whole assembly (Scheme 1). Interestingly, in all cases, at least one of the internal copper atoms displays different coordination geometry with respect to the other external ones, along with a conspicuous conformational change of the organic ligands that fill its coordination sphere. Importantly, this conformational change renders the ligand's NH moieties available to establish additional hydrogen bonding (HB) interactions, which, as it will be shown, are important to allow for the catenation of the two [2 × 2] grids in **4** and **5**. In addition to this, while in the case of **4** and **5** we observed a tetrahedral coordination geometry for one of the internal copper ions, which led us to assume a Cu(ii)-to-Cu(i) transformation,^9^ in **6**, both internal copper ions are instead found to be penta-coordinated. Indeed, the versatility of the coordination of the Cu(ii)/Cu(i) couple appears to be fundamental for the formation of the [2]-catenane species, since the use of Zn(ii) salts only led to more trivial [2 × 2] grid-like structures, such as **3**. To the best of our knowledge, structures **4–6** represent a unique set of [2]-catenane systems including the heterometallic grid catenanes **5** and **6**, which are the first of their kind.

## Results and discussion

Compound **1** (Scheme 1) can be easily synthesized by the addition of hydrazine to the sodium salt of dimethyl 5-sulfoisophthalate in MeOH.^10^

**Fig. 1:**
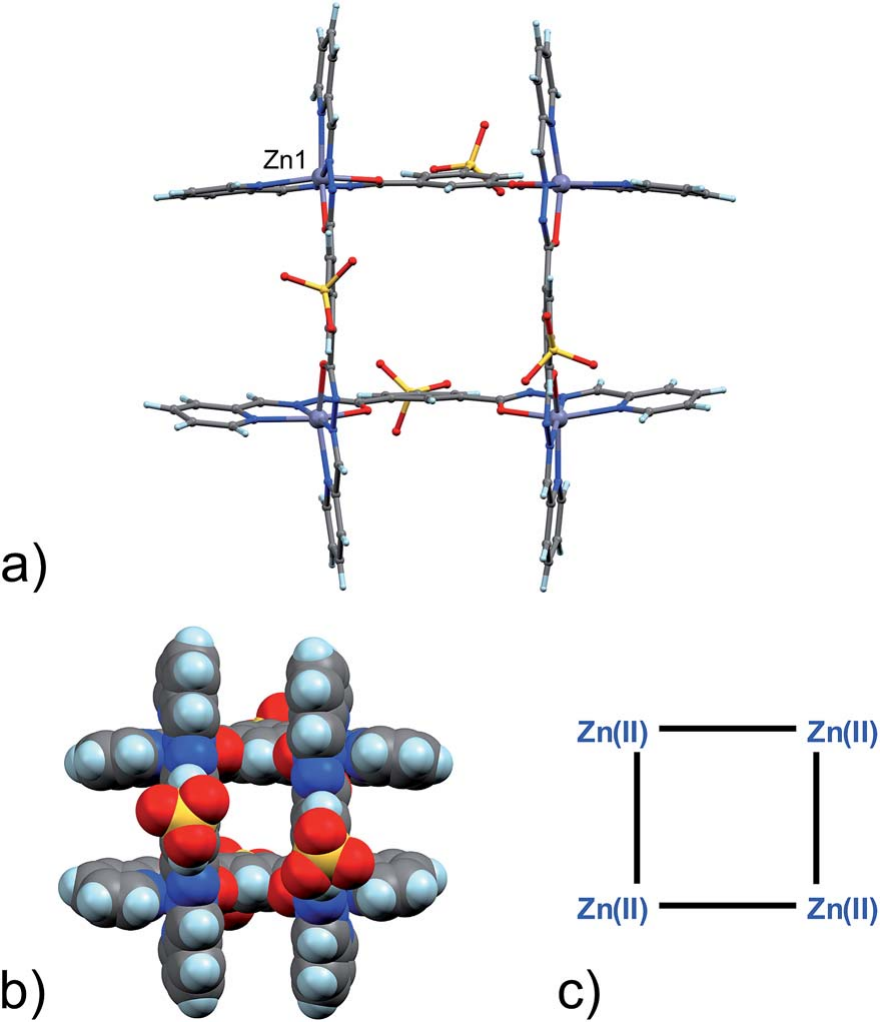
(a) Ball and stick and (b) space-filling model of **3**. (c) Schematic representation of **3**. C: gray, H: turquoise, N: blue; O: red, S: yellow, Zn: violet.

The slow evaporation of a 1 : 2 : 1 mixture of **1**, 2-formylpyridine, **2**, and Zn(ii) acetate in a 2 : 1 H_2_O : DMF mixture produced good quality yellow single crystals of **3** after approximately one week. Their X-ray diffraction analysis revealed the formation of the [2 × 2] grid shown in Fig. 1.

**Scheme 2:**
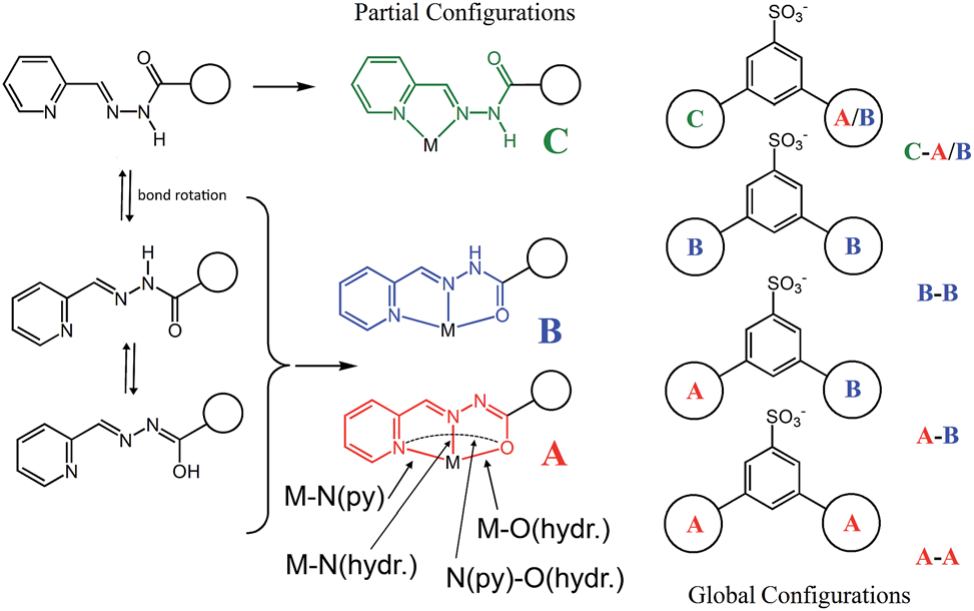
A schematic representation of the possible partial, A, B, C, and global configurations that can be adopted by the organic framework upon metal coordination within the grid **3** and the [2]-catenane systems **4–6**. Relevant distances such as M–N(py), M–N(hydr.) and M–O(hydr.) are also defined.

Four Zn(ii) cations are held in place in a square grid-like arrangement by four ditopic ligands, each generated by Schiff base condensation of **1** with two equivalents of **2**, and each interacting in a tridentate fashion with two metals. The latter ions are found to be in an octahedral environment, coordinated by four nitrogen and two oxygen atoms. As far as the ligand is concerned, it is important to note that tridentate coordination can be attained by different configurations, depending on the degree of ligand deprotonation. In Scheme 2, the possible configurations for the metal binding site are shown. While both configurations A and B lead to a tridentate NNO coordination mode, deprotonation occurs in A. On the other hand, configuration C results in a NN bidentate coordination. On the whole, each ligand can be found in a combination of A–A, A–B, B–B and A/B–C global configurations, either symmetric or not, all of which are chemically feasible and have been observed in metal complexes with similar ligands.^11^ In particular, coordinating C–O bond lengths are expected to show a change upon (de)protonation *viz.*, C–O^–^ (A) *vs.* C=O (B), with the latter being shorter than the former. However, in grid **3**, due to high symmetry and possible positional disorder, the relevant C–O distance, but also C–N and N–N distances, are found to be averaged out and thus the exact configuration status of the ligand in the grid-like structure cannot be directly determined by the X-ray diffraction data. Component **1** is expected to be a sodium salt and we were unable to find and model any metallic counter-ions in the lattice. This may be simply due to the high symmetry and disorder present in the structure, which prevented us from clearly identifying and differentiating between counterions and solvent. However, we notice that if all ligands adopted configuration A–B, with each ligand being mono-deprotonated, **3** would be neutral on the whole, with no additional counter-cations needed to balance the charge. Indeed, this would represent a quite simple and appealing hypothesis that we are poised to believe as real, yet unconfirmed.

Packing of the [2 × 2]-grids in **3** can be interpreted as directed by steric factors and by the polarity of the hydrophilic sulfonate groups (Fig. S5, ESI†). [2 × 2] grids of this kind are quite common in the literature and have also been obtained with ligands of similar chemical structures.^7^

**Fig. 2:**
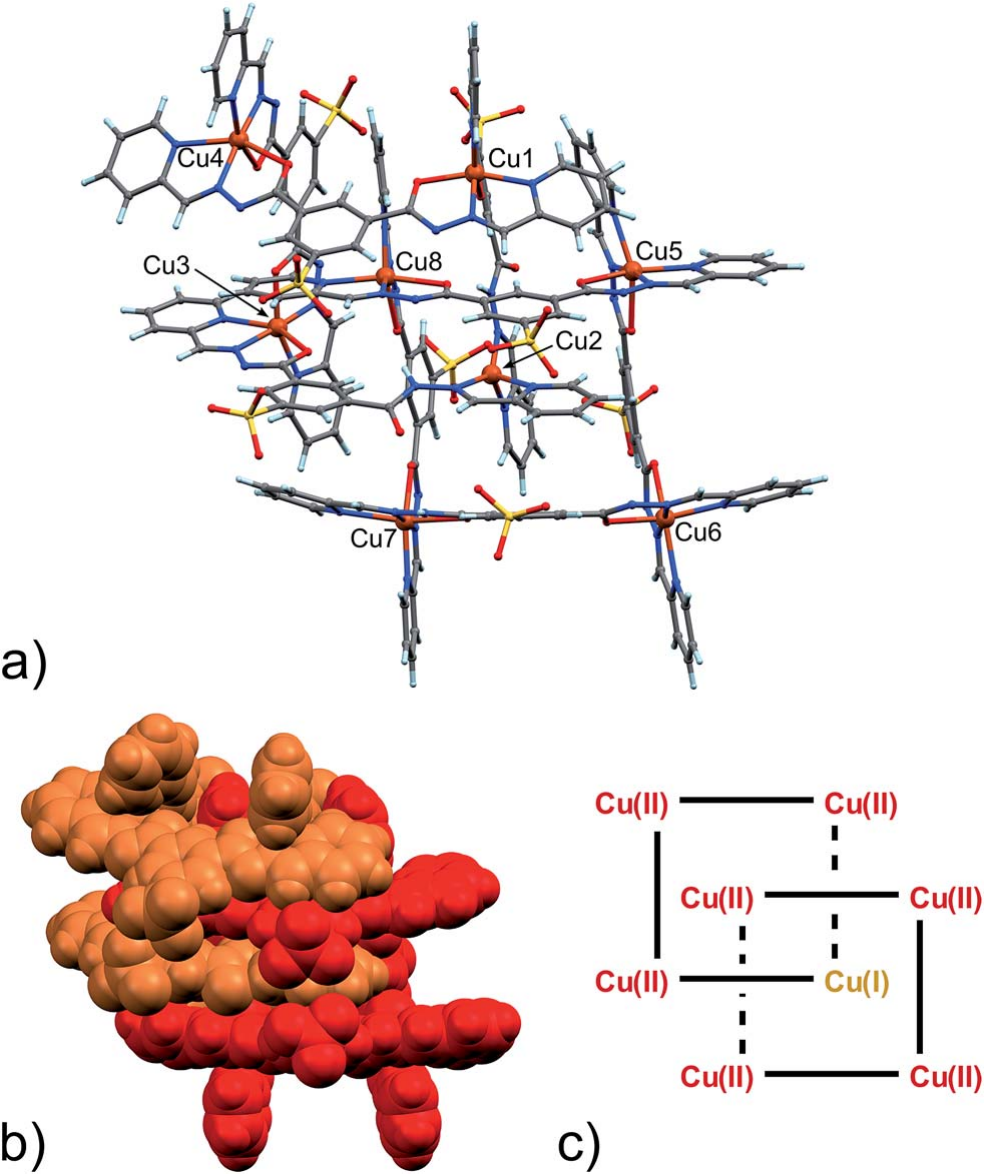
(a) Ball and stick and (b) space-filling model of **4**. (c) Schematic representation of **4**. C: gray, H: turquoise, N: blue; O: red, S: yellow, Cu: orange.

It is well known that changing the identity of the metal ions can results in a complete change of coordination geometry and, hence, of the final complex architecture.^12^ As to our system, interestingly, the replacement of Zn(ii) ions with Cu(ii) ions led to a quite surprising outcome. Indeed, the slow evaporation of a 1 : 2 : 1 mixture of **1**, **2** and Cu(OAc)_2_ in a 2 : 1 mixture of H_2_O/DMF solution produced good quality single crystals of [2]-catenane **4**, whose X-ray determined structure is shown in Fig. 2.

As clearly seen,^13^ [2 × 2] grids are formed also in this case. However, two grids are interlocked with each other to form a [2]-catenane superstructure. The two concatenated grids are not symmetrical and one is slightly larger than the other, with the distances between adjacent copper ions in the 8.359–8.665 Å and 8.549–9.054 Å range for the smaller and larger grid, respectively. The concatenation leads to a rotation of the two grids with respect to each other of approximately 40°. The detailed inspection of its structural elements reveals that not all of the copper ions adopt the same coordination geometry. Indeed, one of the two internal copper ions is found to be tetra-coordinated, while the other seven are in octahedral environments. The two ligands that bind this “anomalous” copper ion display a non-symmetrical structure with one of their binding sites having a different configuration (C in Scheme 2), and acting as a NN bidentate ligand. This coordination motif, as well as the hydrogen bonding around the internal Cu(ii) atoms, implies that deprotonation is not occurring on those sites of the ligands. Since tetrahedral coordination of Cu(ii) with NN-bidentate ligands is not common and the crystallization conditions are known to induce reduction of Cu(ii),^9^ we propose that a Cu(ii)-to-Cu(i) transformation is occurring in this case.

The coordination geometry of most of the other Cu(ii) atoms shows the characteristic distortion from an ideal octahedron due to the Jahn–Teller effect (Table S7, ESI†). This feature, while confirming the oxidation state of Cu ions as +2, will also become important later.

As far as charge state is concerned, also in this case, as in the previously described Zn(ii)-grid **3**, we were not able to find and model any additional counter-cations in the lattice (neither Na^+^, nor Cu^2+^/Cu^+^ ions), possibly implying the neutrality of the whole structure. If that were the case, the presence of a lower valence state for the Cu(i) ion would be therefore counterbalanced, for example, by one of the eight ligands forming the [2]-catenanes adopting a fully protonated state.

Inter-macrocycle interactions, together with the necessity of efficient packing, are usually responsible for the formation of concatenated architectures. In this case, the two ligand sites that act as NN-bidentate do not make use of their carbonyl groups in the coordination with the metal and two additional inter-grid NH···O=C interactions are thus established (N_24A_–H_24A_···O_11H_ (2.933(8) Å, ang. 167°) + C_17A_–H_17A_···O_11H_ (3.184(10), ang. 133°) (bifurcated); N_9B_–H_9B_···O_22G_ (2.833(9) Å, ang. 164°) + C_17B_–H_17B_···O_22G_ (3.160(10) Å, ang. 140°) (bifurcated)). This reminded us of the well-known Vögtle–Hunter type of [2]-catenane,^14^ where inter-macrocyclic interactions between amidic NH and carbonyl groups were first observed. Notably, the ligand C-type partial configuration is stabilized by the presence of weak intramolecular hydrogen bonds (C_26A_–H_26A_···O_22A_ (2.751(10) Å, ang. 126°), C_7B_–H_7B_···O_11B_ (2.796(13) Å, ang. 119°)). Additional structural stabilization is surely provided by the abundant water forming a network of hydrogen bonds, which, however, remain undefined due to the less-than-ideal data quality and a large degree of disorder.

The [2]-catenane units pack with direct interactions by means of N–H···O hydrogen bonds, supported by weaker C–H···O hydrogen bonds, with sulfonate groups, forming centrosymmetric pairs. The same can be observed for the other, symmetrically independent catenane species.

To the best of our knowledge, there exists only one example of [2]-catenane systems made by interlocked grids.^8^ In that case, a 1 : 1 Co(ii)/Co(iii) metal distribution within the grid was observed due to the oxidation of Co(ii) to Co(iii) by air. The ligand employed displayed two different conformations in the grid structure, however, no configuration change was observed, making our system unique.

Intrigued by the unexpected grid concatenation observed in **4** and by the different behaviour of Zn(ii) and Cu(ii) under these experimental conditions, we wondered if the process could be selective and we tried to crystallize a mixture of **1** and **2** with Cu(ii) and Zn(ii) salts in a 1 : 2 : 0.5 : 0.5 ratio. After several crystallization attempts, we were able to obtain good quality single crystals of two species, **5** and **6** (Scheme 1). They are both heterometallic species and while displaying strong similarities with **4**, are still substantially different. Such mixed valence heterometallic [2]-catenane species made of grid units are unprecedented.

**Fig. 3:**
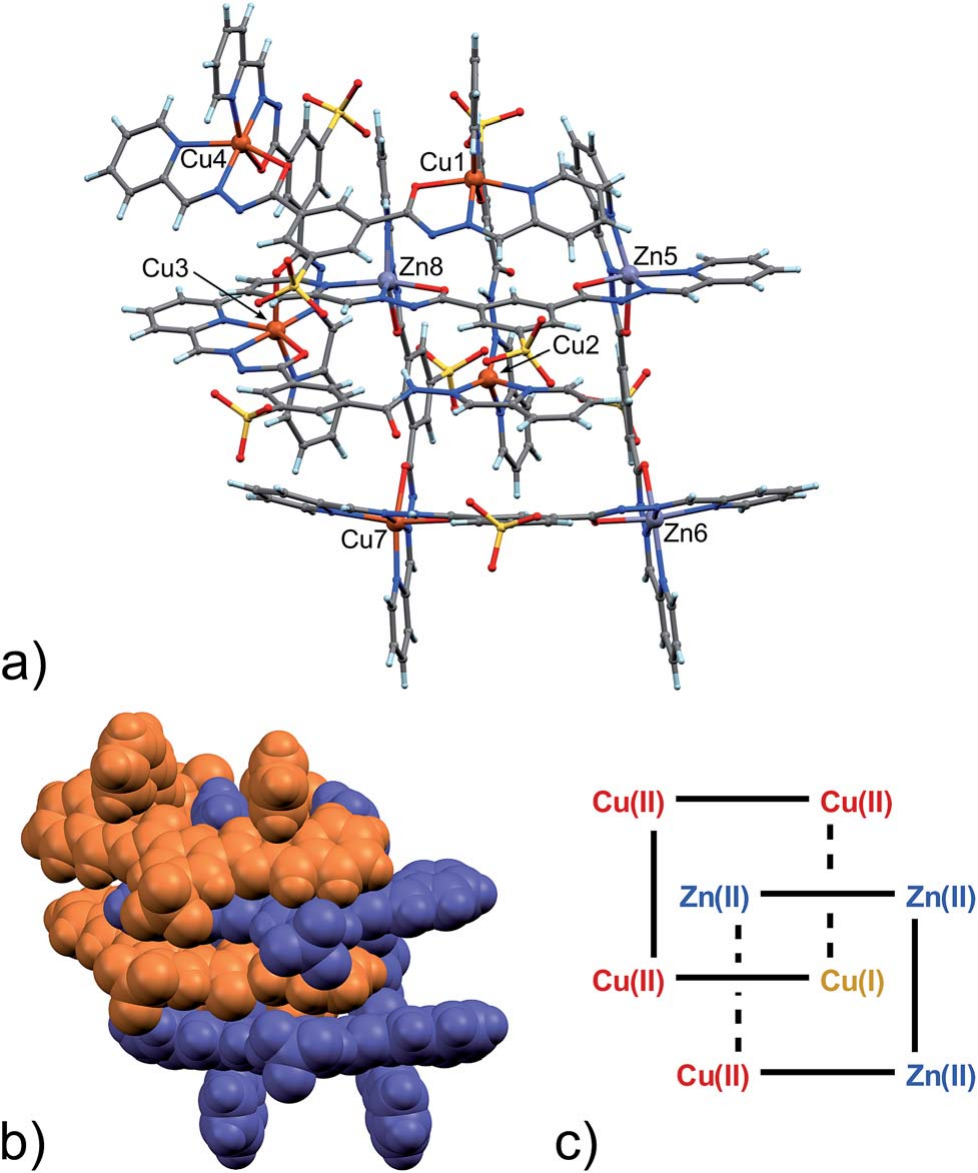
(a) Ball and stick and (b) space-filling model of **5**. (c) Schematic representation of **5**. C: gray, H: turquoise, N: blue; O: red, S: yellow, Zn: violet, Cu: orange.

With the assignment of metal identity being of exceeding importance in this work, we devoted particular attention to establishing a set of criteria^15^ that could be used to properly distinguish Zn(ii) from Cu(ii) in **5** (Fig. 3). First of all, the internal metal ion that displays a tetrahedral coordination environment was unambiguously assigned as Cu(i). As far as discriminating Zn(ii) from Cu(ii) ions, the observation of Jahn–Teller effect – present in d^9^ electronic configuration as in Cu(ii) ions but not in d^10^ Zn(ii) ions – was very helpful. Indeed, in **5**,^13^ four metal ions display such an effect, which leads to the elongation of the metal–ligand bond distances in the apical coordination positions. For example, the mean apical Cu(ii)–N(py) and Cu(ii)–O(hydr.) distances in the catenane unit of **5** discussed here of 2.26 and 2.32 Å, respectively, can be compared to the respective equatorial distances of 2.06 and 2.05 Å.

The availability of monometallic Zn(ii) grid **3** and the Cu(ii)/Cu(i) [2]-catenane **4** provided a solid ground for the comparison with **5**, aimed at the discrimination between copper and zinc centres. Also, an analysis of the CSD for Zn(ii) and Cu(ii) complexes with similar ligands provided additional confirmation on the validity of the criteria chosen and allowed us to evaluate the effect of the strain due to the [2]-catenane complex formation on the coordination geometries around the metal ions.^16^ Indeed, in simple Cu(ii) and Zn(ii) metal complexes, the M–N(hydr) distance (Scheme 2) seems to be the clearest indicator of the metal atom type, with the average distances for Zn(ii) being larger – in cases of both A and B type configuration – than those for Cu(ii). Furthermore, we considered the N(py)–O(hydr) distance (Scheme 2) as revealing for the presence of Jahn–Teller effect. Indeed, its mean values are larger for Zn complexes than for Cu ones and distributed over a narrower interval (4.087–4.357 Å, to be compared with the 3.850–4.539 Å interval found in the case of Cu-complexes, see ESI† – CSD search).

In the [2]-catenane systems, strain due to concatenation is surely present and the divergence between the average Cu(ii) and Zn(ii) coordination environments is less profound.^17^ However, it has been always possible to determine the Zn(ii)/Cu(ii) quite confidently (Table S8, ESI†).

Packing in **5** is essentially very similar to that found in **4**, showing interdigitation of the neighbouring catenane units (Fig. S9 in ESI†). However, an additional solvated Zn(ii) ion is found in the lattice. The latter ion, present as a DMF/water solvate, balances the overall charge. This feature again confirms the considerable adaptability of the ligand, which can adjust to the environment in terms of global charge distribution.

In any case, given the central importance of determining the metal composition in **5**, we sought additional confirmation of the Zn/Cu ratio in **5**. Energy-dispersive X-ray spectroscopy (EDS) and atomic absorption spectrophotometry (AAS) analyses made on the same single crystals analysed by X-ray diffraction brought in comforting results. EDS data show a Zn : Cu ratio = 0.63 on average of multiple measurements (see ESI†). This ratio is in perfect agreement with the Zn : Cu ratio provided by the metric analysis on the X-ray diffraction data, also taking into account the presence of an additional Zn(ii) ion (with 0.4 occupancy factor, total 6.4 : 10 Zn : Cu equals to 0.64) in the lattice (see ESI†).^18^ Moreover, the AAS of Cu–Zn catenane **5** gives 5.87 (±0.01%) for Cu and 3.63 (±0.07%) for Zn, giving a ratio of 0.62 ± 0.01, in accordance with the EDS and the X-ray analysis made on **5**.

An important aspect that is common to both **4** and **5** pertains to the observed necessity of a change in the coordination geometry of, at least, one the internal metal ions in order to produce the concatenated structures. Steric factors can be involved, however, we must stress that the tetra-coordination exerted on the Cu(i) ion by the C-type configuration ligands frees two carbonyl groups per [2]-catenane unit, and they can then be involved in stabilizing inter-grid interactions. In the case of structures **4** and **5**, each grid unit is connected to the concatenated one by two such NH···O=C HB interactions.

**Fig. 4:**
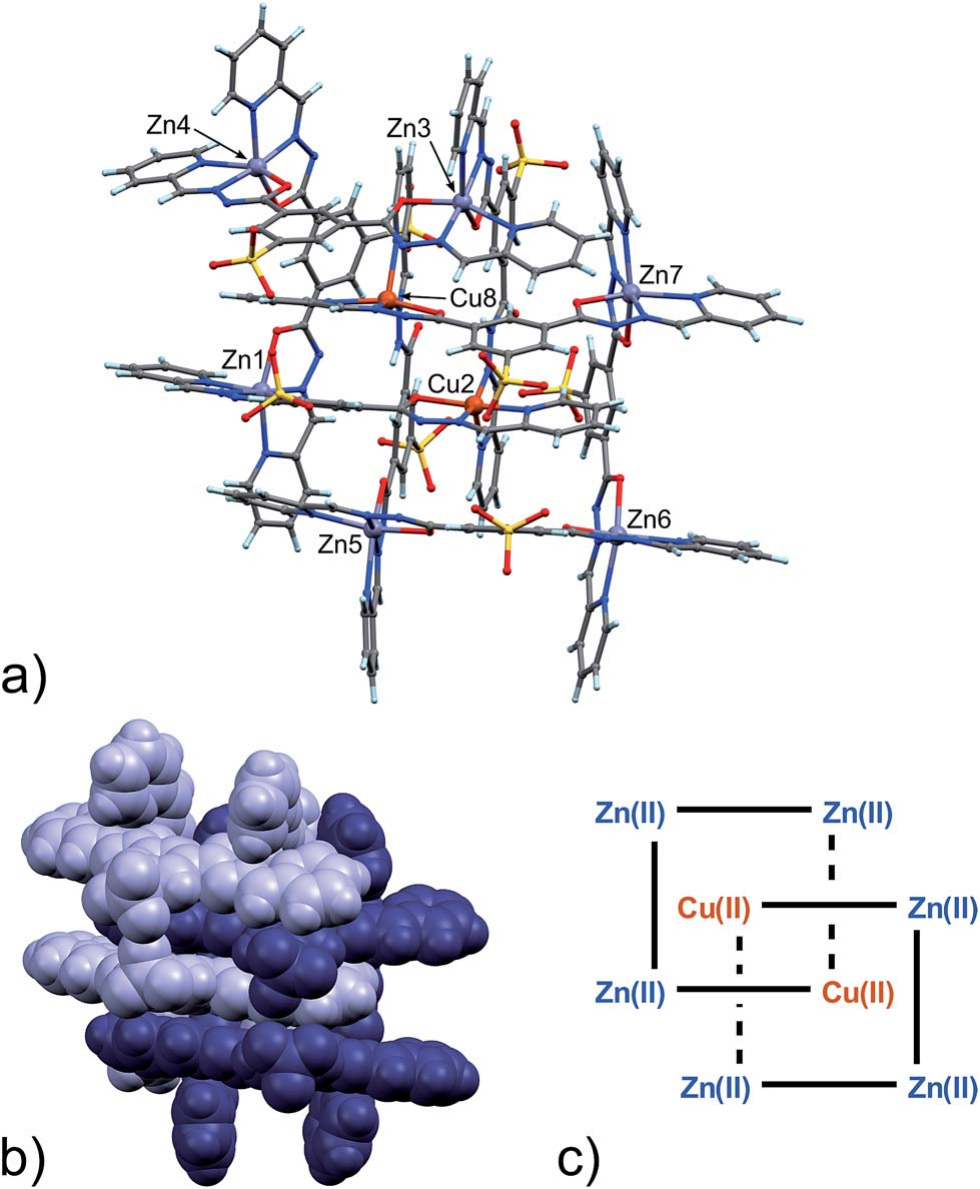
(a) Ball and stick and (b) space-filling models of **6**. (c) Schematic representation of **6**. C: gray, H: turquoise, N: blue; O: red, S: yellow, Zn: violet, Cu: orange.

From the data above, it might seem that the concatenation process has a slight preference for the incorporation of copper over zinc, however, the attainment – by a slightly modified crystallization procedure – and characterization of **6** disproves this hypothesis. Indeed, the crystal structure of **6**, shown in Fig. 4, features a [2]-catenane structure having six Zn(ii) metals and only two Cu(ii) ions.^13^ Again, similarly to the situation found in **4** and **5**, the catenation leads to a change in the configuration of the ligands involved in the coordination of the internal Cu(ii) ions. This strongly suggests that the catenation happens in the presence of at least one internal copper atom and a change in the configuration of the ligands bound to it. In the case of **6**, the symmetrical grids require both of the internal Cu(ii) ions to be penta-coordinated (Table S9, ESI†), whereas in the structures of **4** and **5**, only one tetra-coordinated internal Cu(i) suffices for catenation. Confident assignment of the metal identity was achieved by a careful metric analysis of the data in relation to **4** and **5**,^19^ and by the comparison with published structures of NNO–NN penta-coordinated Cu(ii) complexes (ESI†).^20^

**Fig. 5:**
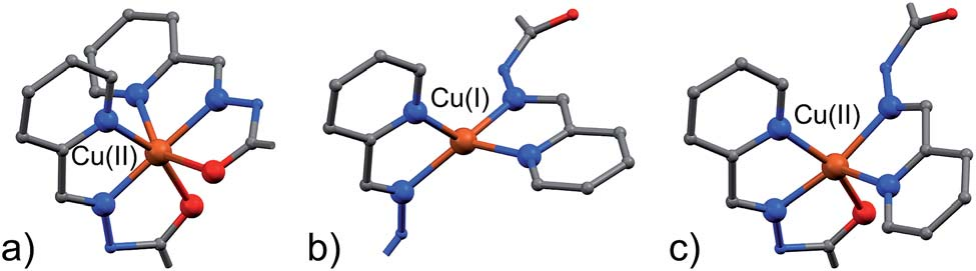
The different coordination modes that Cu(ii) and Cu(i) adopt in the [2]-catenane structures. (a) (NNO)_2_ hexa-coordinated Cu(ii), as in **4** and **5**; (b) internal (NN)_2_ tetra-coordinated Cu(i) as in **4** and **5**; internal (NNO)(NN) penta-coordinated Cu(ii) as in **6**; C: gray, N: blue; O: red, S: yellow, Cu: orange. Hydrogen atoms are omitted for clarity.

A visual summary of the three different environments that the Cu(ii/i) metal ions adopt in the structurally similar [2]-catenanes **4–6** is shown in Fig. 5. Hexa- and penta-coordinated Cu(ii), and tetra-coordinated Cu(i) are indeed observed.

Twelve Na^+^ counter-cations can be located and quite appropriately modelled in the structure (six for each [2]-catenane adduct, there are two of them in the asymmetric unit),^21^ therefore, on average, each grid must be triply negative and the four ligands wholly bear 11 negative charges.

Interestingly, in this case, neither of the non-coordinating carbonyl groups (C_10H_ =O_11H_, C_10B_ =O_11B_), freed by the change of configuration of the ligand, are involved in inter-grid interactions as in **4** and **5**, but instead participate in binding to Na^+^ ions mediated by water molecules.

It is important to add here that the crystals of **6** were obtained by a slightly modified crystallization procedure, that is, by ethanol vapour diffusion into a 1 : 1 H_2_O : DMF solution. Since the organic components of the [2]-catenanes are quite adaptable both in terms of their conformation and configuration, the combination of ligand and copper ion gives access to a large configurational and conformational space, and the crystallization of a different structure does not come as a surprise. Clearly, the difference in the experimental procedure (evaporation *vs.* anti-solvent diffusion) presented a significant enough perturbation to lead to the formation of a completely different structure in **6**, with all the distinguishing features as already highlighted above. As a final comment, we note that complexes **4–6** were obtained in yields close to 30%, a figure which is far from optimal, and in striking variance with other reported systems, which can be obtained in significantly higher yields, and sometimes even quantitatively.^5a,b^

## Conclusion

Structural complexity is sought by chemists to refine their synthetic capabilities and to discover new properties. This work describes three examples of [2]-catenane systems made of interlocked metal–organic grids obtained by multicomponent self-assembly, and characterized in the solid state by single crystal X-ray diffraction. These unique metal complex systems are either mono- (**4**) or hetero-metallic (**5** and **6**) and display multivalence copper (**4** and **5**). To the best of our knowledge, **5** and **6** represent the first catenated heterometallic grids reported to date. Their apparent heterogeneity should not be misleading. Indeed, all the [2]-catenane systems possess common features. For example, at least one of the internal metal ions is copper, which displays a different coordination geometry with respect to the other external ones, along with a conspicuous conformational change of the organic ligand involved. The role of the Cu(ii)/Cu(i) metal pair, especially in terms of its versatility, is here highlighted as a key factor responsible for the formation of such species. We would also like to emphasize that the presented work provides a simple route to obtain octanuclear complexes, some of which are non-symmetric, and with defined metal composition, which would be otherwise unaccessible. This feature stems from the particular environment surrounding the internal metal ions within the framework and from the catenation event. The possibility to lay a given metal ion into a precise position in such a complex structure could have indeed a large impact in the development of functional materials.

Further research is aimed at verifying the degree of control that can be attained over the systems in terms of overall charge distribution in the [2]-catenanes and final Zn/Cu ratio within the hetero-metallic species. Also, structural modifications of components **1** and **2** have been envisaged in order to improve control over metal identities and complex stoichiometry. Extensive crystallization trial experiments with a wider range of d-block metal salts and concentrations are currently ongoing in our laboratories.
