# Supplementary material for: Mixed valence mono- and hetero-metallic grid catenanes
Source: Chem Sci. 2015 Jun 30;6(10):5712–8. doi: 10.1039/c5sc01851j (PMC5512231; doi:10.1039/c5sc01851j)
Supplement: Supplementary file 1 [file SC-006-C5SC01851J-s001.pdf]

Electronic Supplementary Material (ESI) for Chemical Science.
This journal is © The Royal Society of Chemistry 2015

ELECTRONIC SUPPLEMENTARY INFORMATION

for

Mixed Valence Mono- and Hetero-Metallic Grid Catenanes

Chandan Giri,[a] Filip Topić,[a] Massimo Cametti[b] and Kari Rissanen[a]**

[a] Department of Chemistry, Nanoscience Center, University of Jyväskylä, P.O. Box 35, Jyväskylä, Finland;
[b] Department of Chemistry, Materials and Chemical Engineering "Giulio Natta", Politecnico di Milano, Via L. Mancinelli 7, 20131 Milan, Italy.

Table of Content

1. CSD search on Cu and Zn complexes with acylhydrazone ligands....................S2

2. Examples of pentacoordinated Cu(II) ions with acylhydrazone ligands....................S6

3. Relevant metric information on the metal-coordination in **3**, the packing of **3**....................S7

4. Relevant metric information on the metal-coordination in **4**, **5** and **6**....................S8

5. Additional content of asymmetric units and packing in **4**, **5** and **6**. S11

6. EDS analysis....................S14

7. Crystallographic tables for **3-6**....................S16

1. CSD search on Cu and Zn complexes with acylhydrazone ligands

In order to facilitate and support the assignation of metal cation type (Zn or Cu) and ligand protonation state, a CSD survey was undertaken. Having in mind that the metal centres of interest predominantly exhibit octahedral coordination by two tridentate ligands, we paid special attention to such coordination mode.

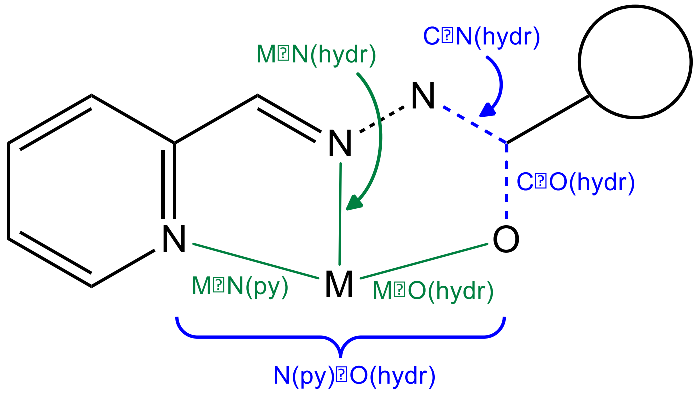

Figure S1. Analysed bond lengths and other distances.

Two possibilities for the tridentate coordination of 2-formylpyridinehydrazone ligand to Cu or Zn were envisaged, namely the coordination of the deprotonated (**Fig. S2a**) and the protonated (**Fig. S2b**) form. The CSD search was designed so as to look for one or the other form, *i.e.* excluding the other completely, in order to yield meaningful average values of the relevant geometrical parameters.

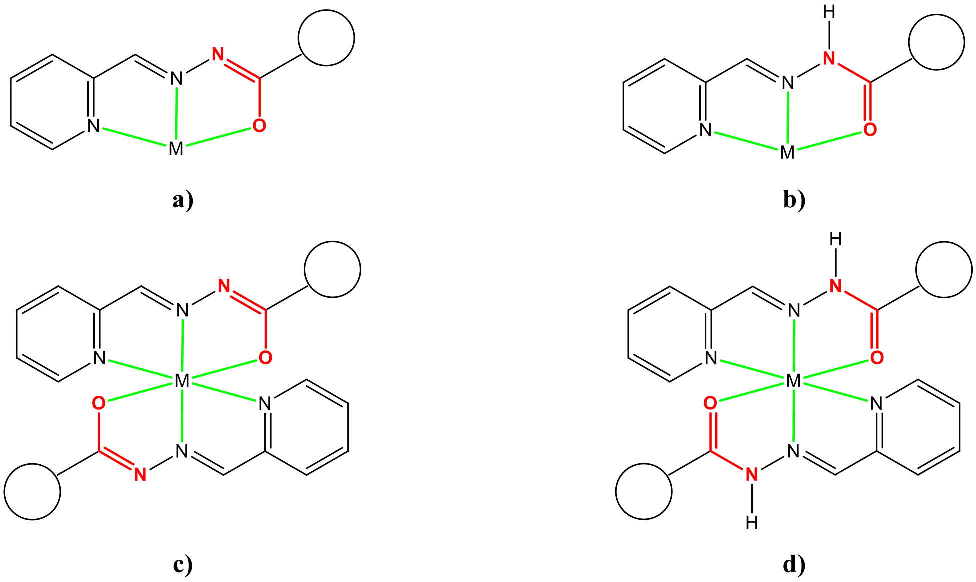

Figure S2. Tridentate coordination by the 2-formylpyridinehydrazone moiety **L** in its **a)** deprotonated and **b)** protonated form. Octahedrally coordinated M = Cu/Zn with two of either **c)** deprotonated or **b)** protonated **L** moieties.

The searches were performed as follows:

1) Cu and Zn complexes were analysed separately. No atoms other than Cu or Zn were permitted in the structures.

2) For each of these, a separate search was performed for either deprotonated or protonated **L** moieties, respectively, with the other form always being excluded completely.

3) Finally, a search was performed for octahedral complexes of either Cu or Zn where the metal would be coordinated by two of either protonated or deprotonated **L** moieties.

Searching for the tridentate coordinating *deprotonated* (**Fig. S2a**) ligand form in complex with zinc yielded 21 structures (**Table S1a**), of which one structure (HABBUH) was omitted as an outlier in two different categories, possibly due to the intramolecular hydrogen bonding in which the hydrazone moiety is involved. On the other hand, a search for the tridentate coordinating *protonated* (**Fig. S2b**) ligand form in complex with zinc yielded 18 structures (**Table S1b**), none of which were omitted, although one of the C···O(hydr) bond length in one of them (ATOREG) seemed to be somewhat of an outlier. Moreover, we found 16 structures (**Table S2a**) where zinc was coordinated by two *deprotonated* **L** moieties (**Fig. S2c**), while only 6 structures (**Table S2b**) were found where zinc was coordinated by two *protonated* **L** moieties (**Fig. S2d**).

Searching for the tridentate coordinating *deprotonated* (**Fig. S2a**) ligand form in complex with copper yielded 109 structures (**Table S3a**), whereas searching for the tridentate coordinating *protonated* (**Fig. S2b**) ligand form in complex with copper yielded 21 structure (**Table S3b**). Furthermore, we found 22 structures (**Table S4a**) where copper was coordinated by two *deprotonated* **L** moieties (**Fig. S2c**), while only 3 structures (**Table S4b**) were found where copper was coordinated by two *protonated* **L** moieties (**Fig. S2d**).

This very basic analysis seems to provide a lot of useful data on the behaviour of ligands possessing moiety **L** with both Zn and Cu cations.

Table S1. Average, minimum and maximum values of the characteristic geometric parameters in the structures of Zn complexes containing **a)** *deprotonated* or **b)** *protonated* moiety **L**.

**Distance**	Zn−N(py)	Zn−N(hydr)	Zn−O(hydr)	N(py)···O(hydr)	C···O(hydr)	C···N(hydr)
**a)** Average (Å)	2.236(72)	2.045(32)	2.129(30)	4.207(62)	1.279(18)	1.330(23)
Minimum (Å)	2.119	1.968	2.057	4.087	1.252	1.275
Maximum (Å)	2.448	2.097	2.211	4.357	1.315	1.381
**b)** Average (Å)	2.147(25)	2.103(26)	2.206(51)	4.170(53)	1.231(11)	1.364(16)
Minimum (Å)	2.100	2.043	2.135	4.062	1.194	1.332
Maximum (Å)	2.208	2.149	2.336	4.279	1.248	1.391

Table S2. Average, minimum and maximum values of the characteristic geometric parameters in the structures of Zn complexes coordinated by two moieties **L** which are both **a)** *deprotonated* or **b)** *protonated*.

**Distance**	Zn−N(py)	Zn−N(hydr)	Zn−O(hydr)	N(py)···O(hydr)	C···O(hydr)	C···N(hydr)
**a)** Average (Å)	2.243(73)	2.042(32)	2.130(31)	4.212(65)	1.279(18)	1.330(25)
Minimum (Å)	2.119	1.968	2.057	4.087	1.252	1.275
Maximum (Å)	2.448	2.097	2.211	4.357	1.315	1.381
**b)** Average (Å)	2.135(23)	2.082(20)	2.170(26)	4.140(33)	1.231(16)	1.357(17)
Minimum (Å)	2.100	2.043	2.135	4.094	1.194	1.332
Maximum (Å)	2.190	2.109	2.228	4.200	1.248	1.382

First, let us focus on the behaviour of Zn(II) with **L**-type ligands (**Table S1**). While the Zn−N(py) distance is longer than the Zn−O(hydr) distance with *deprotonated* **L**, this is reversed for *protonated* **L**. These two distances can thus serve as an indicator of the protonation state of the ligand. Furthermore, C···O(hydr) bond (see **Fig. S1**) distance also changes markedly when **L** is *protonated*, exhibiting a 0.05 Å shortening, whereas the C···N(hydr) bond (see **Fig. S1**) distance shortens for around 0.03 Å. Finally, it is interesting to note the ESD values of the mean Zn−O/N distances. Since the ESD on the mean bond distance for Zn−N(hydr) seems not to change significantly relative to the protonation state of the ligand, it can serve as a reference in discussing the ESDs on mean bond distances for Zn−N(py)and Zn−O(hydr). Thus, upon *deprotonation* of **L** the ESD for the mean Zn−N(py) distance more than doubles, while the ESD for the mean Zn−O(hydr) distance remains very similar to the one for Zn−N(hydr). However, with *protonated* **L**, the opposite is observed: ESD for the mean Zn−O(hydr) distance doubles, with the ESD for the mean Zn−N(py) distance remains very similar to the one for Zn−N(hydr). We believe this is the result of the Zn−O(hydr) bond being stronger than Zn−N(py) with *deprotonated* **L**, whereas with *protonated* **L**, the Zn−N(py) is stronger. All the described trends are also valid for the narrowed-down dataset, where only structures containing Zn octahedrally coordinated with two **L** moieties were analysed (**Table S2**).

Table S3. Average, minimum and maximum values of the characteristic geometric parameters in the structures of Cu complexes containing a **a)** *deprotonated* or **b)** *protonated* moiety **L**.

**Distance**	Cu−N(py)	Cu−N(hydr)	Cu−O(hydr)	N(py)···O(hydr)	C···O(hydr)	C···N(hydr)
**a)** Average (Å)	2.072(88)	1.924(33)	2.043(105)	4.030(159)	1.283(22)	1.323(23)
Minimum (Å)	1.951	1.815	1.932	3.850	1.210	1.230
Maximum (Å)	2.397	2.051	2.399	4.532	1.344	1.401
**b)** Average (Å)	2.080(96)	1.972(34)	2.142(131)	4.094(189)	1.243(13)	1.367(10)
Minimum (Å)	1.979	1.930	1.994	3.908	1.213	1.347
Maximum (Å)	2.300	2.062	2.460	4.539	1.271	1.380

Table S4. Average, minimum and maximum values of the characteristic geometric parameters in the structures of Cu complexes coordinated by two moieties **L** which are both **a)** *deprotonated* or **b)** *protonated*.

**Distance**	Cu−N(py)	Cu−N(hydr)	Cu−O(hydr)	N(py)···O(hydr)	C···O(hydr)	C···N(hydr)
**a)** Average (Å)	2.141(105)	1.929(46)	2.121(127)	4.153(190)	1.277(27)	1.322(26)
Minimum (Å)	1.994	1.815	1.932	3.884	1.210	1.230
Maximum (Å)	2.397	2.015	2.399	4.532	1.329	1.382
**b)** Average (Å)	2.144(30)	1.972(23)	2.184(54)	4.212(68)	1.234(10)	1.375(4)
Minimum (Å)	2.085	1.950	2.084	4.086	1.226	1.371
Maximum (Å)	2.167	2.016	2.234	4.272	1.254	1.380

Next, the behaviour of the Cu(II) with L-type ligands is examined (**Table S3**). Again, it seems that with *deprotonated* ligand, the Cu−O(hydr) distance is shorter than Cu−N(py), whereas the situation is reversed when **L** is *protonated*. As expected, the Cu−N(hydr) distance also increases when L is *protonated*. Similarly, C···O(hydr) bond is about 0.04 Å longer for the *deprotonated* form, whereas in the same form the C···N(hydr) distance is roughly 0.04 Å shorter than in the *protonated* one. Furthermore, the ESD values once

again seem rather informative. The ones for mean Cu−N(py) and Cu−O(hydr) bond lengths are 3-4 times higher than those for Cu−N(hydr), which we believe is a result of the pronounced Jahn-Teller distortion causing large variation among these values, while not affecting the Cu−N(hydr). Similar trends are also observed for the narrowed-down dataset concerning the Cu(II) coordinated by two deprotonated **L** ligands (**Table S4a**). However, it is difficult in this case to discuss the average parameters for the narrowed-down dataset in case of Cu(II) coordination by two protonated ligands (**Table S4b**), since it only contains 3 structures.

Finally, it is certainly interesting to compare the behaviour of the analysed complexes depending on the nature of the cation [Zn(II) or Cu(II)]. Thus, we can note that on average, all the coordination bonds are longer in case of Zn(II) as compared to Cu(II) (possibly a result of d^{10} vs. d^9 electron configuration), as well as the expected absence of Jahn-Teller effect in the Zn(II) complexes. Concerning the coordination bond lengths, the M−N(hydr) seems to be the clearest indicator of the metal atom type, with the average distances for Zn(II) being larger than 2 Å for both protonated and deprotonated ligand, whereas the average distances for Cu(II) are under 2 Å. Furthermore, N(py)···O(hydr) distance indicates the presence of Jahn-Teller effect. First, their mean values are larger for Zn complexes than for Cu complexes. Moreover, for Zn complexes, they are distributed over a narrower interval (4.087-4.357 Å) than in case of Cu complexes (3.850-4.539 Å), which is all in line with the presence of Jahn-Teller effect for Cu.

2. Examples of pentacoordinated Cu(II) ions with acylhydrazone ligands

Table S5. Relevant distances for pentacoordinated Cu(II) ions in light of their similarity with our systems.

	Cu–N(py)	Cu–N(hydr)	Cu–O(hydr)	N(py)···O(hydr)
LIBKIQ	2.064(3)	1.899(4)	2.035(3)	3.998(4)
	2.193(4)	1.999(4)	-	-
SACWUP	2.019(2)	1.923(3)	2.017(2)	3.964(3)
	2.172(2)	2.026(2)	-	-
	Cu–N(py)	Cu–N(hydr)	Cu–O(hydr)	N(py)···O(hydr)
WUJDAH	2.0157(16)	1.9228(15)	1.9871(13)	3.937(2)
	”Cu–N(py)”	”Cu–N(hydr)”		
	1.9947(15)	2.3768(14)	-	-
	Cu–N(py)	Cu–N(hydr)	Cu–O(hydr)	N(py)···O(hydr)
WUJDEL	1.998(3)	1.930(3)	1.985(3)	3.902(4)
	”Cu–N(py)”	”Cu–N(hydr)”		
	2.180(3)	2.000(3)	-	-

from J. A. K. Howard *et al.*, *Inorg.Chem*. 2004, **43**, 4278-4288 (see Table 10, SACWUP)

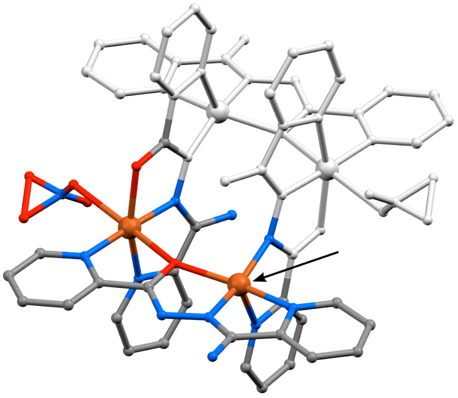

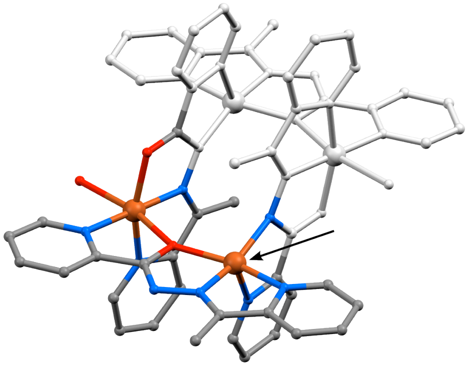

from J. A. K. Howard *et al.*, *Inorg. Chem.* 1999, **38**, 5266-5276 (see Table 10, LIBKIQ)

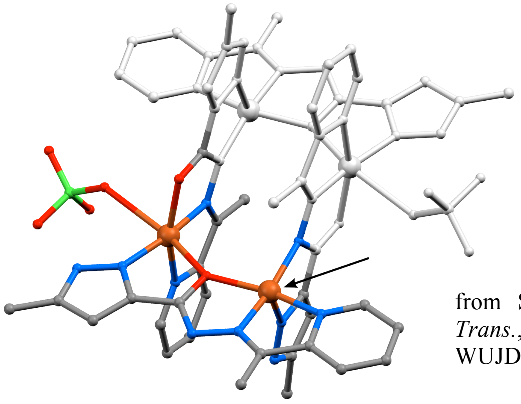

from S. K. Kar *et al.*, *Dalton Trans.*, 2009, 8215-8226
WUJDEL (left), WUJDAH right)

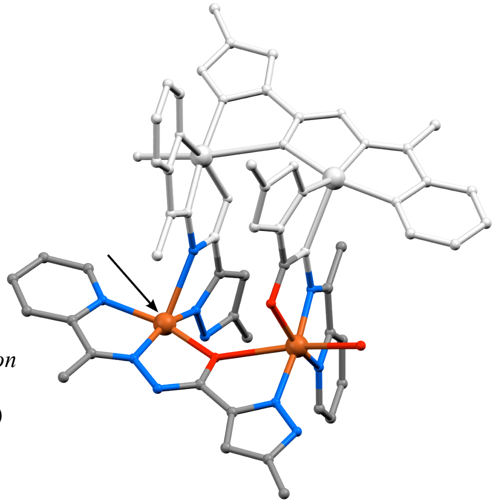

Figure S3. Reproduction of the reported pentacoordinated Cu(II) ions in light of their similarity with our system. The relevant copper atoms are marked with arrows.

3. Relevant Metric Information on the Metal-Coordination in 3, the packing of 3

Table S6. Relevant Metric Information on the Metal-Coordination in **3**.

		M−N(py)		M−N(hydr)		M−O(hydr)	N(py)···O(hydr)		C···O(hydr)		C···N(hydr)
Zn1	N1A	2.239(3)	N8A	2.063(4)	O11A	2.096(3)	4.178(4)	C10A	1.257(8)	N9A	1.338(9)
Zn1^i	N32A^i	2.197(3)	N25A^i	2.064(4)	O22A^i	2.099(3)	4.134(4)	C23A^i	1.285(9)	N24A^i	1.360(9)
$i = 3/4 - y, - 1/4 + x, 3/4 - z$											

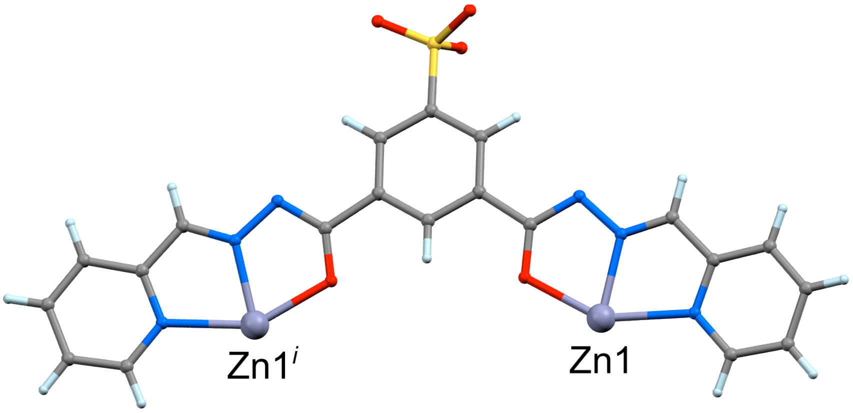

Figure S4. Asymmetric unit of Zn(II)-grid complex **3**.

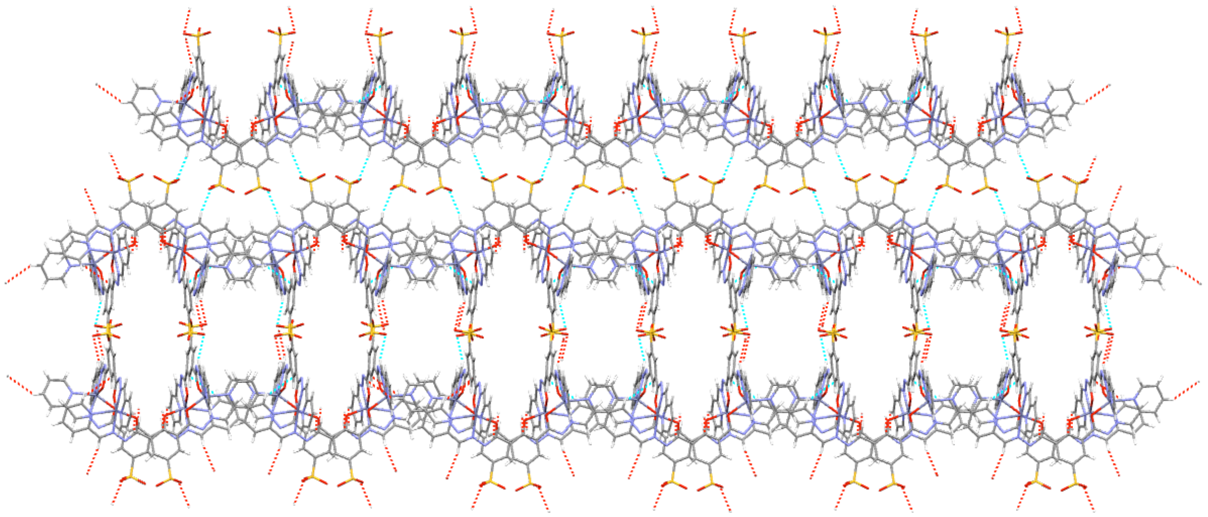

Figure S5. Packing of Zn(II)-grid complex **3**.

4. Relevant Metric Information on the Metal-Coordination in 4, 5 and 6

Table S7. Relevant Metric Information on the Metal-Coordination in **4**

		M−N(py)		M−N(hydr)		M−O(hydr)	N(py)···O(hydr)		C···O(hydr)		C···N(hydr)
Cu1	N32D	2.035(6)	N25D	1.927(6)	O22D	1.992(5)	3.960(7)	C23D	1.280(9)	N24D	1.350(10)
	N1A	2.321(5)	N8A	2.019(6)	O11A	2.350(5)	4.486(7)	C10A	1.222(9)	N9A	1.366(9)
Cu2	N32A	2.023(7)	N25A	2.024(8)	O22A	-	-	C23A	1.222(11)	N24A	1.364(10)
	N1B	2.042(8)	N8B	1.998(8)	O11B	-	-	C10B	1.242(12)	N9B	1.351(13)
Cu3	N32B	2.250(7)	N25B	1.999(8)	O22B	2.348(5)	4.436(9)	C23B	1.233(10)	N24B	1.352(11)
	N1C	2.041(7)	N8C	1.909(8)	O11C	2.047(6)	4.015(9)	C10C	1.264(10)	N9C	1.353(11)
Cu4	N32C	2.051(6)	N25C	1.919(8)	O22C	2.002(5)	3.990(8)	C23C	1.275(10)	N24C	1.325(10)
	N1D	2.298(7)	N8D	2.012(6)	O11D	2.361(5)	4.476(9)	C10D	1.223(10)	N9D	1.358(10)
Cu5	N32H	2.125(8)	N25H	1.957(10)	O22H	2.128(6)	4.145(10)	C23H	1.227(12)	N24H	1.343(13)
	N1E	2.152(7)	N8E	2.002(10)	O11E	2.223(7)	4.223(10)	C10E	1.258(12)	N9E	1.345(13)
Cu6	N32E	2.239(8)	N25E	2.002(10)	O22E	2.249(7)	4.320(11)	C23E	1.233(13)	N24E	1.344(13)
	N1F	2.110(8)	N8F	1.936(10)	O11F	2.072(8)	4.096(11)	C10F	1.285(14)	N9F	1.318(16)
Cu7	N32F	2.276(8)	N25F	2.011(8)	O22F	2.430(7)	4.510(10)	C23F	1.208(11)	N24F	1.381(11)
	N1G	2.011(7)	N8G	1.918(8)	O11G	1.991(6)	3.941(9)	C10G	1.240(10)	N9G	1.362(10)
Cu8	N32G	2.140(6)	N25G	1.977(7)	O22G	2.209(5)	4.191(8)	C23G	1.279(9)	N24G	1.290(11)
	N1H	2.184(6)	N8H	2.007(7)	O11H	2.256(5)	4.278(7)	C10H	1.243(9)	N9H	1.326(11)
Cu9	N32L	2.026(11)	N25L	1.959(14)	O22L	2.036(8)	3.996(13)	C23L	1.275(16)	N24L	1.345(16)
	N1I	2.260(8)	N8I	2.009(11)	O11I	2.342(9)	4.441(12)	C10I	1.254(19)	N9I	1.332(17)
Cu10	N32I	2.030(11)	N25I	2.044(11)	O22I	-	-	C23I	1.223(13)	N24I	1.324(15)
	N1J	2.016(9)	N8J	2.046(11)	O11J	-	-	C10J	1.255(16)	N9J	1.312(16)
Cu11	N32J	2.267(7)	N25J	2.022(8)	O22J	2.287(8)	4.382(11)	C23J	1.242(13)	N24J	1.341(14)
	N1K	2.059(6)	N8K	1.918(8)	O11K	2.024(6)	4.004(8)	C10K	1.286(10)	N9K	1.340(10)
Cu12	N32K	2.042(9)	N25K	1.900(11)	O22K	2.070(7)	4.038(11)	C23K	1.299(14)	N24K	1.326(14)
	N1L	2.264(8)	N8L	2.001(8)	O11L	2.304(8)	4.393(11)	C10L	1.251(13)	N9L	1.346(12)
Cu13	N32P	2.029(9)	N25P	1.928(10)	O22P	2.004(8)	3.961(12)	C23P	1.280(13)	N24P	1.324(15)
	N1M	2.244(8)	N8M	2.008(8)	O11M	2.356(8)	4.420(11)	C10M	1.234(13)	N9M	1.378(13)
Cu14	N32M	2.300(8)	N25M	2.027(8)	O22M	2.398(8)	4.498(11)	C23M	1.235(9)	N24M	1.331(10)
	N1N	2.033(7)	N8N	1.923(8)	O11N	1.990(6)	3.951(9)	C10N	1.286(9)	N9N	1.307(11)
Cu15	N32N	2.180(7)	N25N	2.020(8)	O22N	2.276(8)	4.287(10)	C23N	1.241(13)	N24N	1.342(14)
	N1O	2.097(8)	N8O	1.936(8)	O11O	2.119(6)	4.117(10)	C10O	1.266(10)	N9O	1.348(10)
Cu16	N32O	2.085(7)	N25O	1.949(8)	O22O	2.092(6)	4.073(9)	C23O	1.263(9)	N24O	1.344(11)
	N1P	2.274(7)	N8P	2.033(8)	O11P	2.242(7)	4.319(10)	C10P	1.288(12)	N9P	1.291(12)

Table S8. Relevant Metric Information on the Metal-Coordination in **5**

		M−N(py)		M−N(hydr)		M−O(hydr)	N(py)···O(hydr)		C···O(hydr)		C···N(hydr)
Cu1	N32D	2.036(14)	N25D	1.98(2)	O22D	2.041(19)	4.01(2)	C23D	1.24(3)	N24D	1.34(4)
	N1A	2.255(13)	N8A	2.088(19)	O11A	2.308(18)	4.39(2)	C10A	1.28(3)	N9A	1.35(3)
Cu2	N32A	2.023(15)	N25A	1.84(2)	O22A	-	-	C23A	1.25(4)	N24A	1.35(4)
	N1B	2.015(16)	N8B	1.98(3)	O11B	-	-	C10B	1.28(4)	N9B	1.34(4)
Cu3	N32B	2.230(15)	N25B	2.08(3)	O22B	2.31(2)	4.39(2)	C23B	1.19(4)	N24B	1.41(4)
	N1C	2.089(15)	N8C	1.97(3)	O11C	2.074(16)	4.07(2)	C10C	1.30(4)	N9C	1.37(4)
Cu4	N32C	2.118(15)	N25C	1.99(2)	O22C	2.020(19)	4.05(2)	C23C	1.30(4)	N24C	1.36(3)
	N1D	2.280(15)	N8D	2.05(2)	O11D	2.277(17)	4.40(2)	C10D	1.28(3)	N9D	1.33(4)
Zn5	N32H	2.144(16)	N25H	1.99(2)	O22H	2.15(2)	4.19(3)	C23H	1.16(4)	N24H	1.42(4)
	N1E	2.191(15)	N8E	2.05(3)	O11E	2.168(19)	4.21(2)	C10E	1.24(4)	N9E	1.38(4)
Zn6	N32E	2.187(16)	N25E	2.06(3)	O22E	2.170(19)	4.23(2)	C23E	1.28(4)	N24E	1.35(4)
	N1F	2.219(16)	N8F	2.10(3)	O11F	2.14(2)	4.22(3)	C10F	1.30(4)	N9F	1.32(4)
Cu7	N32F	2.257(16)	N25F	2.01(3)	O22F	2.39(2)	4.47(3)	C23F	1.21(4)	N24F	1.35(4)
	N1G	2.012(16)	N8G	1.91(2)	O11G	2.06(2)	3.99(3)	C10G	1.26(3)	N9G	1.30(4)
Zn8	N32G	2.169(15)	N25G	2.06(2)	O22G	2.168(19)	4.16(2)	C23G	1.28(4)	N24G	1.31(4)
	N1H	2.171(14)	N8H	2.07(2)	O11H	2.16(2)	4.15(2)	C10H	1.28(4)	N9H	1.23(4)
Cu9	N32L	2.069(18)	N25L	1.95(3)	O22L	2.07(2)	4.04(3)	C23L	1.31(4)	N24L	1.41(4)
	N1I	2.268(17)	N8I	2.03(3)	O11I	2.20(2)	4.32(3)	C10I	1.29(5)	N9I	1.34(4)
Cu10	N32I	2.003(15)	N25I	2.04(3)	O22I	-	-	C23I	1.27(5)	N24I	1.34(5)
	N1J	2.010(18)	N8J	1.94(3)	O11J	-	-	C10J	1.33(4)	N9J	1.34(4)
Cu11	N32J	2.255(16)	N25J	2.12(3)	O22J	2.20(2)	4.31(3)	C23J	1.31(4)	N24J	1.26(5)
	N1K	2.104(15)	N8K	1.95(2)	O11K	2.076(19)	4.08(2)	C10K	1.25(4)	N9K	1.33(3)
Cu12	N32K	2.065(18)	N25K	1.91(3)	O22K	2.071(19)	4.05(3)	C23K	1.21(4)	N24K	1.40(4)
	N1L	2.290(17)	N8L	2.06(3)	O11L	2.24(2)	4.38(3)	C10L	1.28(4)	N9L	1.35(5)
Zn13	N32P	2.13(2)	N25P	2.04(3)	O22P	2.08(2)	4.11(3)	C23P	1.25(4)	N24P	1.37(4)
	N1M	2.164(16)	N8M	2.05(3)	O11M	2.30(2)	4.32(2)	C10M	1.22(4)	N9M	1.34(4)
Cu14	N32M	2.262(16)	N25M	2.06(2)	O22M	2.319(19)	4.38(2)	C23M	1.26(4)	N24M	1.30(4)
	N1N	2.086(15)	N8N	1.99(3)	O11N	2.003(19)	4.01(2)	C10N	1.30(4)	N9N	1.32(4)
Zn15	N32N	2.187(12)	N25N	2.05(3)	O22N	2.23(2)	4.26(2)	C23N	1.19(4)	N24N	1.35(4)
	N1O	2.120(16)	N8O	2.04(2)	O11O	2.146(17)	4.15(2)	C10O	1.23(3)	N9O	1.37(3)
Zn16	N32O	2.134(15)	N25O	2.07(3)	O22O	2.088(19)	4.07(2)	C23O	1.29(3)	N24O	1.34(4)
	N1P	2.235(15)	N8P	2.06(3)	O11P	2.12(2)	4.16(2)	C10P	1.36(4)	N9P	1.27(4)

Table S9. Relevant Metric Information on the Metal-Coordination in **6**

		M−N(py)		M−N(hydr)		M−O(hydr)	N(py)···O(hydr)		C···O(hydr)		C···N(hydr)
Zn1	N32D	2.243(7)	N25D	2.021(7)	O22D	2.159(5)	4.261(9)	C23D	1.285(9)	N24D	1.360(9)
	N1A	2.142(7)	N8A	2.034(6)	O11A	2.062(5)	4.084(8)	C10A	1.257(8)	N9A	1.338(9)
Cu2	N32A	2.037(7)	N25A	1.947(5)	O22A	2.032(5)	3.984(8)	C23A	1.290(8)	N24A	1.301(9)
	N1B	2.153(6)	N8B	2.070(5)	O11B	-	-	C10B	1.222(8)	N9B	1.381(8)
Zn3	N32B	2.295(11)	N25B	2.059(7)	O22B	2.119(5)	4.258(12)	C23B	1.256(9)	N24B	1.324(10)
	N1C	2.174(9)	N8C	2.020(7)	O11C	2.097(7)	4.139(11)	C10C	1.250(12)	N9C	1.355(13)
Zn4	N32C	2.235(7)	N25C	2.030(7)	O22C	2.133(6)	4.218(9)	C23C	1.270(11)	N24C	1.304(12)
	N1D	2.195(6)	N8D	2.040(6)	O11D	2.074(6)	4.142(8)	C10D	1.256(9)	N9D	1.362(9)
Zn5	N32H	2.240(7)	N25H	2.061(6)	O22H	2.109(4)	4.199(8)	C23H	1.276(8)	N24H	1.331(9)
	N1E	2.194(7)	N8E	2.043(5)	O11E	2.100(5)	4.155(8)	C10E	1.278(8)	N9E	1.347(9)
Zn6	N32E	2.162(7)	N25E	2.061(7)	O22E	2.077(6)	4.110(9)	C23E	1.263(9)	N24E	1.347(9)
	N1F	2.253(7)	N8F	2.060(7)	O11F	2.124(6)	4.238(9)	C10F	1.274(9)	N9F	1.333(10)
Zn7	N32F	2.183(7)	N25F	2.032(7)	O22F	2.073(6)	4.139(9)	C23F	1.260(10)	N24F	1.329(12)
	N1G	2.226(9)	N8G	2.044(7)	O11G	2.112(6)	4.189(11)	C10G	1.252(9)	N9G	1.371(11)
Cu8	N32G	2.029(7)	N25G	1.919(5)	O22G	2.025(5)	3.967(8)	C23G	1.283(8)	N24G	1.340(10)
	N1H	2.180(6)	N8H	2.029(5)	O11H	-	-	C10H	1.221(8)	N9H	1.357(8)
Zn9	N32L	2.158(7)	N25L	2.025(7)	O22L	2.065(6)	4.107(10)	C23L	1.301(11)	N24L	1.323(11)
	N1I	2.241(8)	N8I	2.046(7)	O11I	2.162(6)	4.246(11)	C10I	1.271(8)	N9I	1.346(12)
Cu10	N32I	2.012(7)	N25I	1.950(5)	O22I	1.961(6)	3.895(9)	C23I	1.288(7)	N24I	1.320(9)
	N1J	2.147(6)	N8J	2.126(6)	O11J	-	-	C10J	1.207(8)	N9J	1.394(9)
Zn11	N32J	2.308(6)	N25J	2.056(6)	O22J	2.125(5)	4.277(8)	C23J	1.277(8)	N24J	1.316(9)
	N1K	2.147(6)	N8K	2.033(6)	O11K	2.085(5)	4.107(8)	C10K	1.292(8)	N9K	1.327(9)
Zn12	N32K	2.201(7)	N25K	2.046(6)	O22K	2.089(6)	4.158(9)	C23K	1.266(9)	N24K	1.347(9)
	N1L	2.215(10)	N8L	2.029(7)	O11L	2.131(6)	4.208(12)	C10L	1.236(9)	N9L	1.326(10)
Zn13	N32P	2.290(7)	N25P	2.053(6)	O22P	2.144(5)	4.277(8)	C23P	1.265(8)	N24P	1.344(9)
	N1M	2.169(6)	N8M	2.025(5)	O11M	2.098(5)	4.140(8)	C10M	1.284(8)	N9M	1.331(8)
Zn14	N32M	2.176(7)	N25M	2.044(6)	O22M	2.085(5)	4.127(8)	C23M	1.243(8)	N24M	1.340(9)
	N1N	2.244(8)	N8N	2.042(6)	O11N	2.125(6)	4.224(9)	C10N	1.258(9)	N9N	1.329(10)
Zn15	N32N	2.150(10)	N25N	2.024(6)	O22N	2.079(6)	4.106(12)	C23N	1.255(9)	N24N	1.365(10)
	N1O	2.255(9)	N8O	2.046(7)	O11O	2.142(6)	4.242(11)	C10O	1.271(9)	N9O	1.313(12)
Cu16	N32O	2.029(9)	N25O	1.918(6)	O22O	2.019(7)	3.978(12)	C23O	1.287(8)	N24O	1.341(10)
	N1P	2.035(6)	N8P	2.286(6)	O11P	-	-	C10P	1.224(9)	N9P	1.359(9)

5. Additional content of asymmetric units and packing in 4, 5 and 6

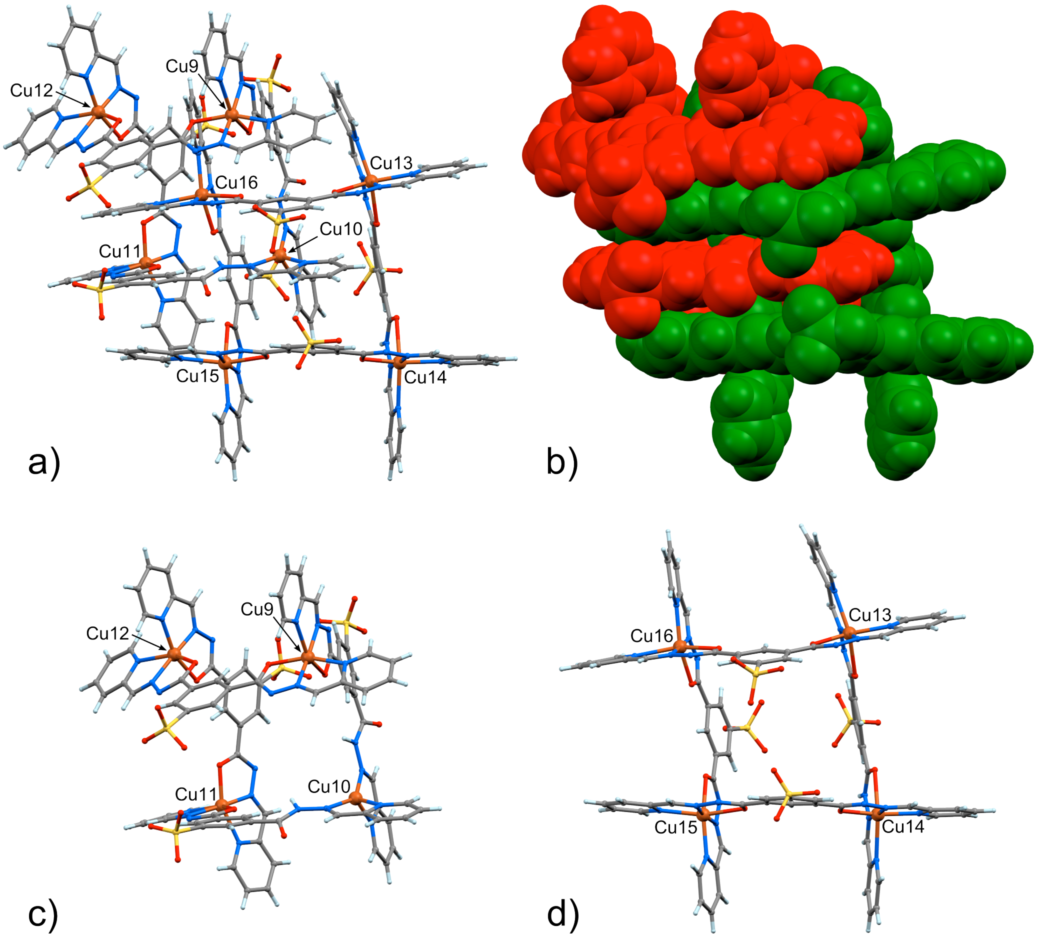

Figure S6. Representations of the other catenane unit present in **4**. a) ball and stick model, b) space-filling model and c, d) catenated grids shown separately.

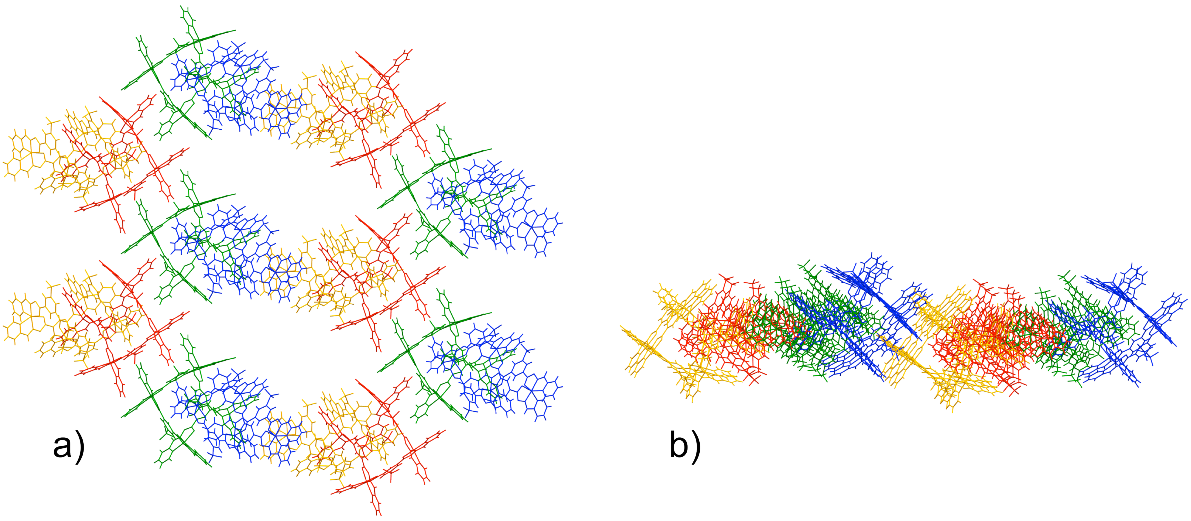

Figure S7. Interdigitation of the catenane units, forming layers, a) from the top and b) from the side. Symmetry independent grids are shown in different colours; red and orange form one and the green and blue form the other catenane unit, respectively.

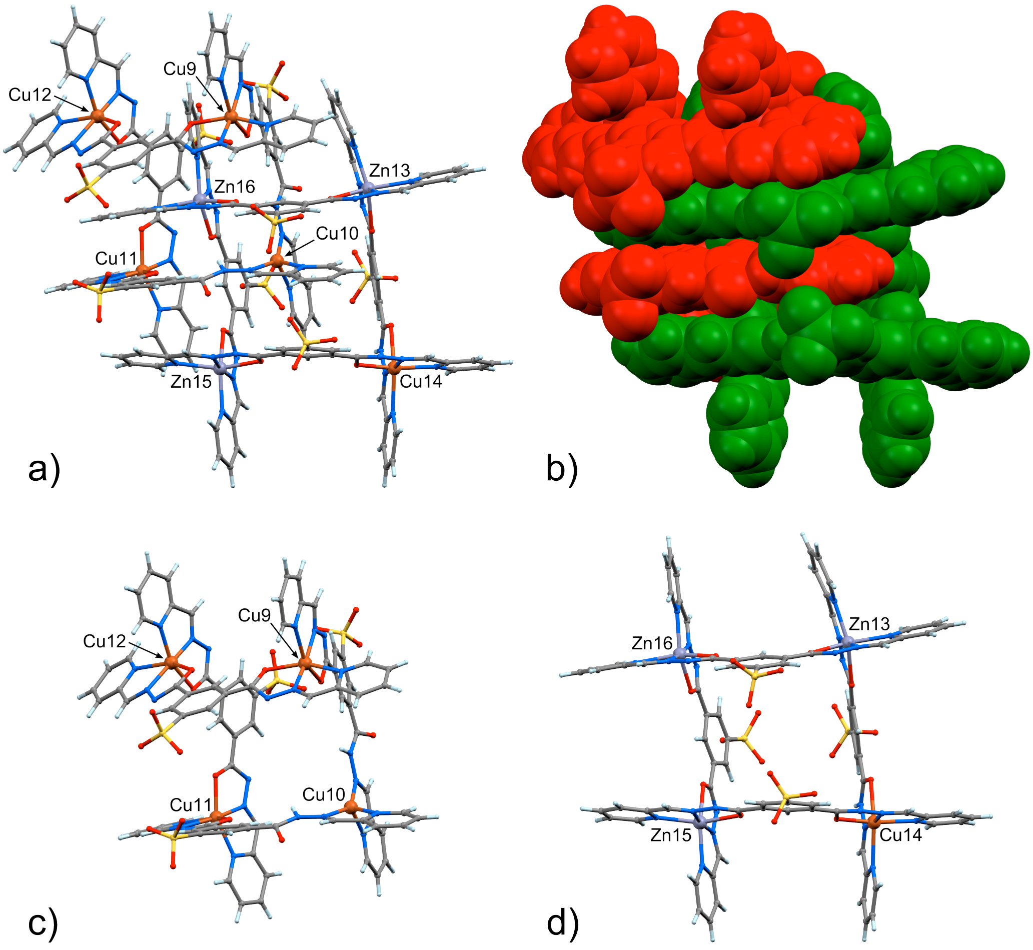

Figure S8. Representations of the other catenane unit present in **5**. a) ball and stick model, b) space-filling model and c, d) catenated grids shown separately.

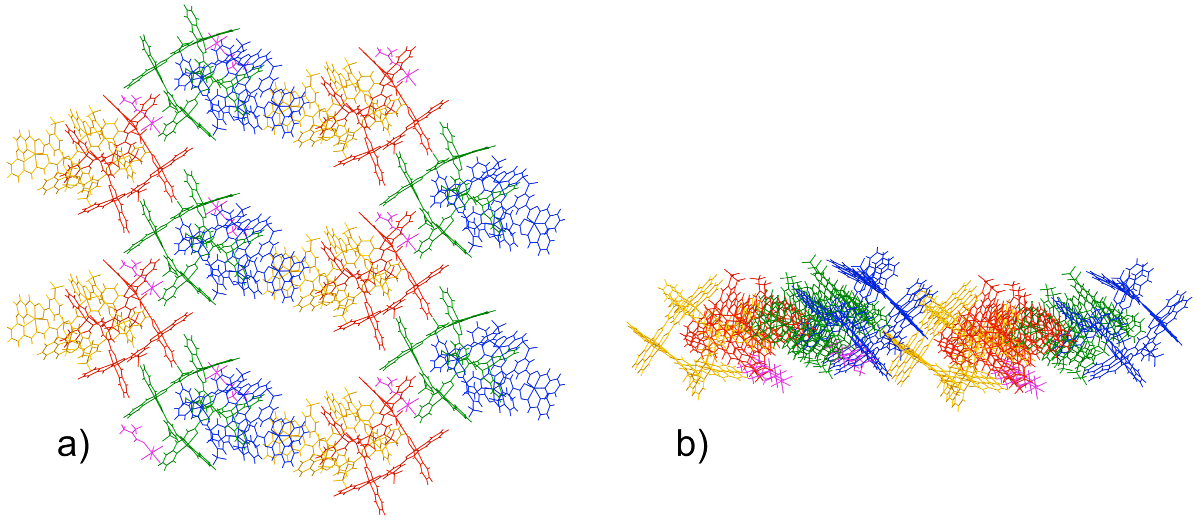

Figure S9. Interdigitation of the catenane units, forming layers, a) from the top and b) from the side. Symmetry independent grids are shown in different colours; red and orange form one and the green and blue form the other catenane unit, respectively. $[Zn(DMF)(H_2O)]^{2+}$ cations are shown in pink.

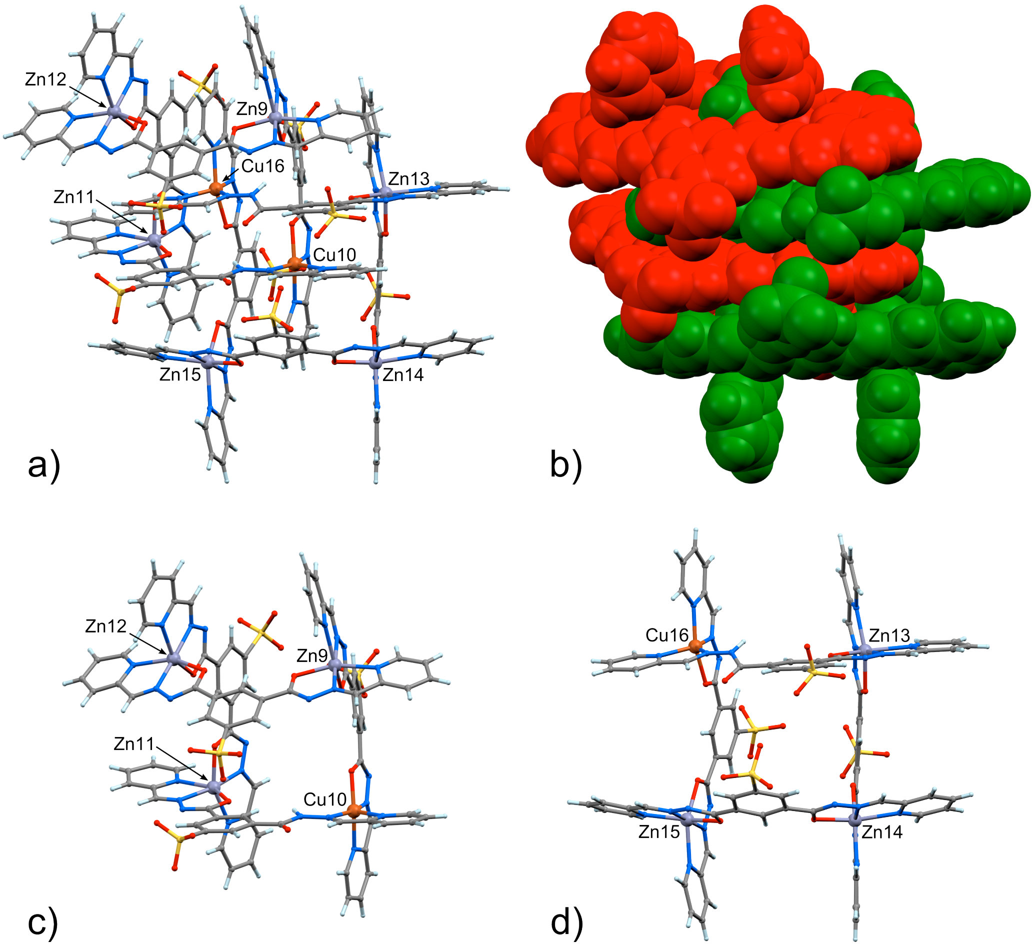

Figure S10. Representations of the other catenane unit present in in **6**. a) ball and stick model, b) space-filling model and c, d) catenated grids shown separately.

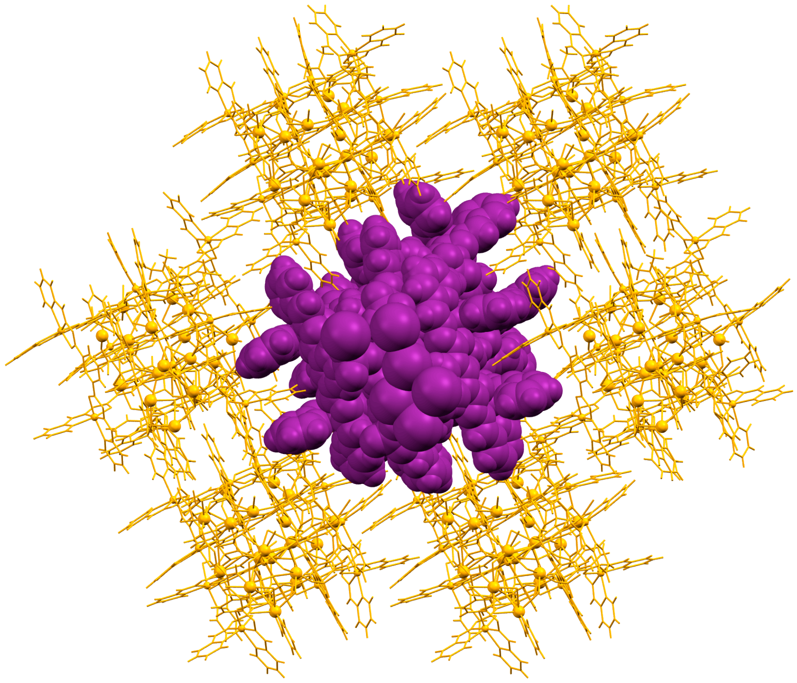

Figure S11. Lateral interdigitation of the Na^{+}-mediated [2]-catenane columnar stacks in **6**.

6. EDS Analysis

To investigate the chemical composition of **3**,**4** and **5**, EDS spectra were measured on the same crystalline material analyzed by XRD. The instrument used was a SEM-EDS Electron Microscope (Field Emission-SEM, FE-SEM, LEO Supra 1535) and the software employed was Software SmartSEM® V05.04 for Field Emission Scanning Electron Microscope (Carl Zeiss NTS GmbH, Oberkochen, Germany). Experimental setup: WD = 8mm, Secondary Electron Detector (SE) 5-20 kV. The crystal samples were still immersed in the oil used as protection during XRD analysis.

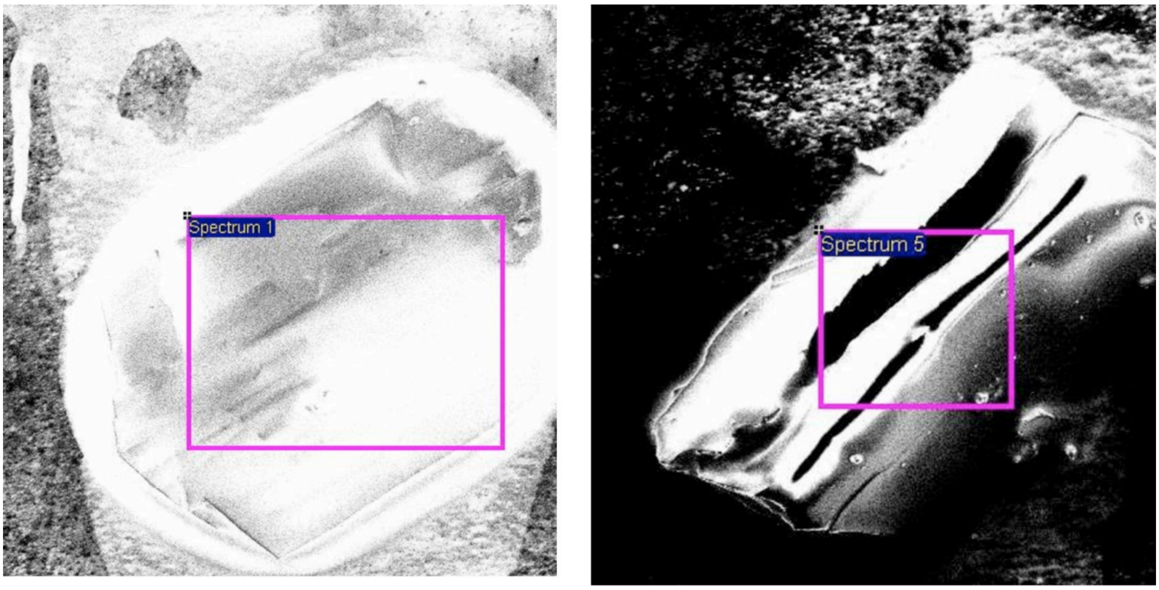

Figure S12. Examples of SEM micrographs with the regions sampled for EDS measurements (purple box).

	Element (shell transition)	Atomic%	Zn/Cu ratio
1	Cu (K)	0.79	0.595
	Zn (K)	0.47	
2	Cu (K)	1.05	0.60
	Zn (K)	0.63	
3	Cu (K)	0.85	0.694
	Zn (K)	0.59	
4	Cu (K)	1.26	0.690
	Zn (K)	0.87	
5	Cu (K)	2.71	0.601
	Zn (K)	1.63	
6	Cu (K)	1.35	0.681
	Zn (K)	0.92	

average Zn/Cu ratio = 0.63

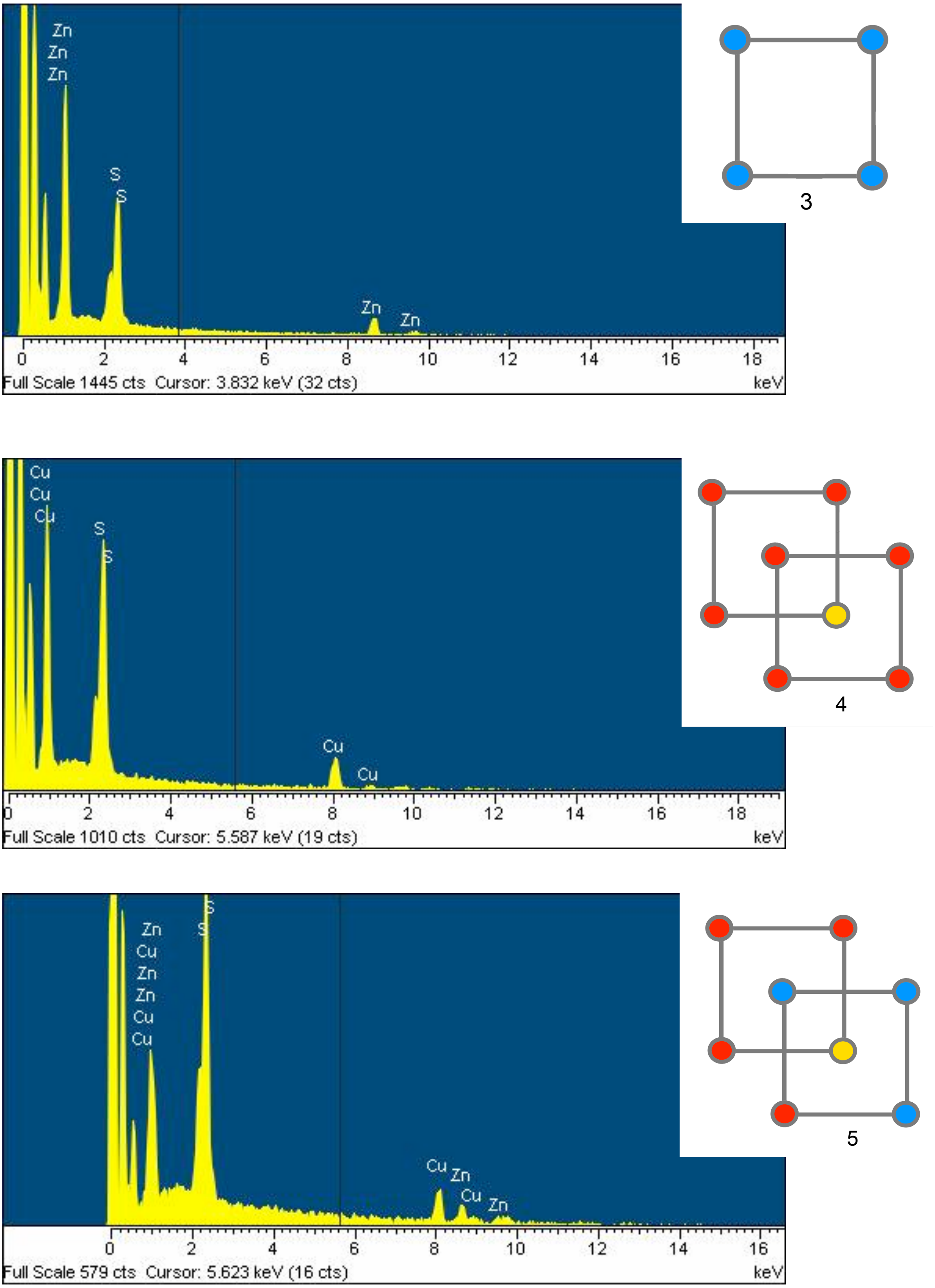

Figure S13. Representative EDS analyses for compounds **3**, **4** and **5**.

7. Crystallographic Tables for structures 3-6

	**3**	**4**	**5**	**6**
CCDC No.	1054512	1054511	1054510	1054509
empirical formula	$C_{80}H_{52}N_{24}O_{20}S_4Zn_4$	$C_{325.25}H_{227.25}Cu_{16}N_{97.75}O_{116.25}S_{16}$	$C_{326.45}H_{230.05}Cu_{10}N_{98.15}O_{119.55}S_{16}Zn_{6.40}$	$C_{324.95}H_{223.55}Cu_4N_{97.65}Na_{11.50}O_{115.55}S_{16}Zn_{12}$
M_w/g	2059.17	8894.38	9007.15	9160.80
T/K	123.0(1)	123.0(1)	123.0(1)	123.0(1)
λ/Å	0.71073	1.54184	0.71073	1.54184
crystal color, shape	pale green, block	brown, plate	green, needle	brown, plate
crystal size/mm^3	0.25×0.20×0.15	0.268×0.084×0.024	0.40×0.10×0.05	0.239×0.187×0.086
crystal system	tetragonal	triclinic	triclinic	monoclinic
space group	$I4_1/a$	$P\bar{1}$	$P\bar{1}$	Pn
a/Å	16.4641(5)	24.6594(6)	24.6339(15)	21.91216(13)
b/Å	16.4641(5)	26.1086(6)	26.2771(16)	32.64068(17)
c/Å	53.397(2)	38.5294(8)	38.566(2)	35.0386(2)
α/°	90	71.7131(19)	71.189(3)	90
β/°	90	78.2731(18)	77.800(2)	98.2694(6)
γ/°	90	87.7169(19)	87.750(2)	90
V/Å^3	14474.1(10)	23054.1(10)	23086(2)	24800.0(2)
Z	4	2	2	2
ρ_c/gcm^{-3}	0.945	1.281	1.296	1.227
μ/mm^{-1}	0.764	2.157	0.932	2.188
F(000)	4176.0	9026.0	9140.4	9279.4
θ range/°	2.27 to 25.24	3.24 to 67.75	0.57 to 18.85	3.32 to 67.75
completeness to θ_{full}	99.9%	98.3%	99.7%	98.5%
reflections collected	33252	129534	97710	79830
independent reflections	6539 [R(int) = 0.0916]	82140 [R(int) = 0.0513]	36227 [R(int) = 0.1877]	52904 [R(int) = 0.0271]
absorption correction	multi-scan	numerical (gaussian)	multi-scan	numerical (analytical)
max. and min. transmission	0.7456 and 0.6447	1.025 and 0.760	0.7453 and 0.5197	0.856 and 0.694
refinement method	full-matrix least squares on F^2	full-matrix least squares on F^2	full-matrix least squares on F^2	full-matrix least squares on F^2
data/restraints/ parameters	6539/766/351	82140/12093/5364	36227/7963/4721	52904/7042/5353
goodness-of-fit on F^2	1.020	1.118	1.236	1.034
final R indices [$I > 2\sigma(I)$]	R1 = 0.0631, wR2 = 0.1645	R1 = 0.1152, wR2 = 0.3072	R1 = 0.1379, wR2 = 0.3434	R1 = 0.0661, wR2 = 0.1860
R indices (all data)	R1 = 0.0955, wR2 = 0.1828	R1 = 0.1676, wR2 = 0.3677	R1 = 0.2743, wR2 = 0.4375	R1 = 0.0696, wR2 = 0.1931
largest diff. peak and hole (eÅ^{-3})	0.557 and −0.335	1.451 and −2.218	1.199 and −0.658	1.239 and −0.561
